# Rational Design of TiO_2_@g-C_3_N_4_/CNT Composite Separator for High Performance Lithium-Sulfur Batteries to Promote the Redox Kinetics of Polysulfide

**DOI:** 10.3390/nano13243084

**Published:** 2023-12-05

**Authors:** Lingling Dong, Wen Jiang, Kefeng Pan, Lipeng Zhang

**Affiliations:** 1 School of Chemistry and Chemical Engineering, Shandong University of Technology, Zibo 255049, China; 2 School of Materials and New Energy, South China Normal University, Shanwei 516600, China

**Keywords:** lithium–sulfur battery, TiO_2_@g-C_3_N_4_, redox kinetics, modified separator

## Abstract

Lithium–sulfur batteries (LSB) show excellent potential as future energy storage devices with high energy density, but their slow redox kinetics and the shuttle effect seriously hinder their commercial application. Herein, a 0D@2D composite was obtained by anchoring polar nano-TiO_2_ onto a 2D layered g-C_3_N_4_ surface in situ, and a functional separator was prepared using multi-walled carbon nanotubes as a conductive substrate. Due to their long-range conductivity, multi-walled carbon nanotubes make up for the low conductivity of TiO_2_@g-C_3_N_4_ to some extent. A lithium–sulfur battery prepared with a modified separator exhibited excellent long-term cycle performance, a good lithium ion diffusion rate, and rapid redox kinetics. The initial specific discharge capacity of the composite was 1316 mAh g^−1^ at 1 C, and a high specific discharge capacity of 569.9 mAh g^−1^ was maintained after 800 cycles (the capacity decay rate per cycle was only 0.07%). Even at the high current density of 5 C, a specific capacity of 784 mAh g^−1^ was achieved. After 60 cycles at 0.5 C, the modified separator retained the discharge capacity of 718 mAh g^−1^ under a sulfur load of 2.58 mg cm^−2^. In summary, the construction of a heterojunction significantly improved the overall cycle stability of the battery and the utilization rate of active substances. Therefore, this study provides a simple and effective strategy for further improving the overall performance and commercial application of lithium–sulfur batteries.

## 1. Introduction

Traditional lithium-ion batteries (LIBs) can no longer meet the future growing demand for high energy storage [1,2]. Therefore, due to their high energy density (2600 Wh kg^−1^) and theoretical specific capacity (1675 mAh g^−1^), lithium–sulfur batteries are promising using secondary battery energy storage systems that have been widely investigated by researchers [2,3,4]. In addition, lithium–sulfur batteries have the advantages of low cost, high element abundance, and environmental friendliness [5,6]. Therefore, the development of lithium–sulfur batteries is of great significance for the future growth of the energy storage field. However, there are still many bottleneck issues preventing the widespread use of lithium–sulfur batteries. These issues mainly include low sulfur utilization due to the insulation properties of S and the charge–discharge product Li_2_S/Li_2_S_2_ [5,7], volume expansion and contraction (up to 80%) of the S-positive electrode during charge–discharge [6], the serious shuttling effect and the slow redox kinetics of soluble polysulfide, and the short-circuit problem caused by Li dendrite resulting in low coulomb efficiency and self-discharge. These problems have seriously hindered the commercial application of lithium–sulfur batteries.

Researchers have evaluated several strategies to address these problems, such as sulfur cathode main body design modification, binder modification, electrolyte additive optimization, anode lithium metal protection, and separator modification strategies. However, modifying the S cathode main body can reduce the energy density of the battery. Modification strategies on the sulfur cathode side can also be applied to separator modification. In recent years, the separator, which is a key component of lithium–sulfur battery, has attracted much significant attention because of its advantages of simple operation and high efficiency. One downside of contemporary commercial PP separators is that they cannot effectively block the shuttle effect of LiPSs [8,9,10,11]. In contrast, carbon materials that exhibit good conductivity and high specific surface area, such as CNF, CNT, and RGO [12,13,14,15,16], can effectively reduce the shuttle effect of LiPSs. These modified separators act as a physical barrier for LiPS shuttling, as well as “secondary collectors” to further reduce the loss of active S substances. However, the weak interaction of single-carbon materials is not sufficient for the efficient immobilization of polysulfides. Therefore, the combination of polar materials with rich active sites on the carbon base (such as metal oxides [3,17,18], sulfides [19], carbides [20], nitrides [21], etc.) on a carbon substrate has been employed as a strategy for the physical adsorption of polysulfides to improve the overall performance of lithium–sulfur batteries. Among them, strongly polar nano-TiO_2_ has rich active sites, good adsorption properties, and strong catalysis activity for polysulfides, and the size of the TiO_2_ nanoparticles can effectively shorten the diffusion path of Li^+^. In addition, the Ti–O bond of polar TiO_2_ has a good sulfur fixation effect, and the low-coordination Ti metal center exhibits strong adsorption performance for polysulfides. It has been reported that a TiO_2_-modified separator can capture polysulfide and reduce the shuttle effect. Yg et al. employed a TiO_2_ NT/RGO composite to modify a lithium–sulfur separator in order to suppress polysulfide shuttling and improve the electrochemical performance of a lithium–sulfur battery [14]. Wang X et al. used graphite nitride carbon (g-C_3_N_4_) material as the modified functional layer of a lithium–sulfur battery separator. This g-C_3_N_4_ was lightweight, highly porous, had a high specific surface area, and was strongly polar [22]. Due to its C–N heteroatoms, g-C_3_N_4_ containing C–N heteroatoms has strong polarity and a rich active center. Therefore, g-C_3_N_4_ can chemically immobilize and rapidly convert lithium polysulfide. This lithium–sulfur battery had a remarkable polysulfide inhibition effect and demonstrated excellent electrochemical performance [23].

Utilizing a multi-functional conductive layer with adsorption properties and a catalytic synergistic effect is an effective strategy for improving redox kinetics and suppressing the shuttle effect of polysulfides in lithium–sulfur batteries. If a material does not have a satisfactory surface area or sufficient electrical conductivity, a heterojunction can be constructed to make up for these deficiencies. Inspired by the above research, a TiO_2_@g-C_3_N_4_ composite with abundant active sites was prepared through a simple pyrolysis method in this study. This TiO_2_@g-C_3_N_4_ heterojunction catalyst was constructed by anchoring TiO_2_ nanoparticles onto the thin g-C_3_N_4_ surface in situ [2]. Then, interlinked multi-walled carbon nanotubes (MWCNTs) were used to form a conductive skeleton to make up for the conductivity deficiency of the composite. The polysulfide shuttling effect and redox kinetics of the modified separator were studied. A lithium–sulfur battery assembled with this modified separator had excellent rate performance (784 mAh g^−1^ at 5 C), a high Li diffusion rate, and stable cycling performance (519 mAh g^−1^ was retained after 1000 cycles at 1 C, with a capacity attenuation rate per cycle of only 0.07% and a Coulomb efficiency of 99%). The long-term cycle stability under a high sulfur load was also tested. Under a high sulfur load of 2.575 mg cm^−2^, a high specific capacity of 718 mAh g^−1^ was retained after 60 cycles at 0.5 C. These results show that the modified membrane has excellent electrochemical performance. This modification strategy can reasonably improve the shuttle effect and enhance the slow redox kinetics of lithium–sulfur batteries, leading to good long-term performance.

## 2. Materials and Methods

### 2.1. Fabrication of a TiO_2_@g-C_3_N_4_ Composite Separator

TiO_2_@g-C_3_N_4_ composites were prepared through a simple pyrolysis method. To prepare orange-yellow TiO_2_@g-C_3_N_4_ powder, urea and TiO_2_ were mixed at a mass ratio of 10:1. This mixture was annealed at a heating rate of 5 °C min^−1^ at 550 °C for 3 h in a tubular furnace under an Ar atmosphere. To obtain the TiO_2_@g-C_3_N_4_/CNT composite-modified separator, CNT and TiO_2_@g-C_3_N_4_ were mixed with a mass ratio of 8:1 to obtain a uniform slurry. This slurry was coated on one side of a commercial PP separator with a 50 µm scraper and dried at 45 °C for 10 h. Separator discs with diameters of 19 mm were punched and stored in a glovebox. Other separators (MWCNTs/PP and TiO_2_/MWCNTs/PP) were prepared through the same method for comparison.

### 2.2. Preparation of the Nano-S and S/CNT Composite Cathode

Highly crystalline nano-S was prepared through a simple chemical synthesis method (Na_2_S_2_O_3_ + 2HCl = 2NaCl + S + SO_2_ + H_2_O). S, MWCNTs, and PVDF were mixed at a mass ratio of 7:2:1, and this mixture was stirred in a high-speed centrifugal mixer to obtain a slurry. This slurry was coated on an aluminum foil and dried at 60 °C for 12 h, then pressed into disks with a diameter of 12 mm.

### 2.3. Material Characterizations

X-ray diffraction (XRD) patterns were obtained using Cu-Kα radiation to verify the successful preparation of the composite. The morphology, size, and microstructure of the samples were characterized via field emission scanning electron microscopy (FESEM) and transmission electron microscopy (TEM). X-ray photoelectron spectroscopy (XPS) was used to detect the surface chemical states of the composite material. The specific surface area was measured using a porosity analyzer. Ultraviolet–visible spectrophotometry (UV–vis) was used to analyze the adsorption capacity of different materials for Li_2_S_6_. The wettability of the electrolyte on different separators was tested by a video optical contact angle measuring instrument.

### 2.4. Visual Adsorption Experiment of Polysulfide (Li_2_S_6_)

Certain amounts of sulfur and Li_2_S (molar ratio of 5:1) were dissolved in a mixed DOL(dioxolame)/DME(1,2-dimethoxyethane) solution (*v*/*v* = 1/1). This mixture was stirred at 60 °C for 24 h to prepare a Li_2_S_6_ solution with a concentration of 0.2 M. Then, 30 mg of MWCNTs or TiO_2_@g-C_3_N_4_ was added to 3 mL of the Li_2_S_6_ solution, and the adsorption capacity was determined via UV–vis spectrophotometry.

### 2.5. Assembly of Symmetric Cells

The active substance and PVDF (mass ratio of 8:1) were dispersed in NMP to prepare a uniform slurry. This slurry was coated on an aluminum foil and dried at 60 °C for 8 h. Next, circular discs with a diameter of 12 mm were cut from the slurry-coated foil. These pressed discs were dried in an oven at 60 °C for 8 h, then stored in a glovebox. A symmetrical battery was assembled using two electrodes coated with the same material as the cathode and anode of the battery. The two electrodes were assembled into a standard CR2032 button battery. A commercial PP separator (Celgard 2400) was used as a substrate for the modified separator, using 0.2 M of Li_2_S_6_ as the electrolyte.

### 2.6. Contact Angle Test

The contact angles of different modified separators were tested by dropping electrolyte on their surfaces. The electrolyte contained 1 M bis(trifluoromethanesulfony)imide lithium (LiTFSI) in a binary solvent of 1, 2-dimethoxyethane (DME) and 1, 3-dioxolane (DOL) (1:1 by volume) with a LiNO_3_ additive (2.0 wt%).

### 2.7. Electrochemical Characterization

Standard CR2032 button cells were assembled in a glovebox with a pure sulfur cathode, lithium negative electrode, and composite-modified separator. Each battery was stored for 12 h to allow the electrolyte (1.0 M LiTFSI and 0.1 M LiNO_3_) to completely infiltrate the cell. The amount of electrolyte in each battery was about 300 µL. The batteries were charged and discharged at constant current (GCD) from 1.7 V to 2.8 V with a Neware tester. An electrochemical workstation (CHI760E) was used to measure the cyclic voltammetry and electrochemical impedance in the voltage range of 1.7–2.8 V at a scanning rate of 0.1 mV s^−1^. The frequency of the alternating current impedance ranged from 100 kHz to 0.1 Hz. The symmetrical battery was measured in a voltage range of −1.0 to 1.0 V at a sweep speed of 50 mV s^−1^.

CV curves were obtained under different scanning rates (0.1–0.5 mVs^−1^) using the electrochemical workstation. The Randles–Ševčík formula was used to calculate the lithium ion diffusion coefficient, D_Li+_. The Li^+^ conductivity of the modified membrane was calculated using the fitted impedance value of the AC impedance spectrum. The activation energy of the electrochemical reaction was calculated through the Arrhenius formula to characterize the catalytic activity and rapid redox kinetics of the material. The rate performance, charge–discharge curve, and long-term cycle performance of the battery were tested with a Xinwei test system, and the cycle stability and cycle life of the battery were investigated.

## 3. Results

### 3.1. Material Characterization

**Figure 1:**
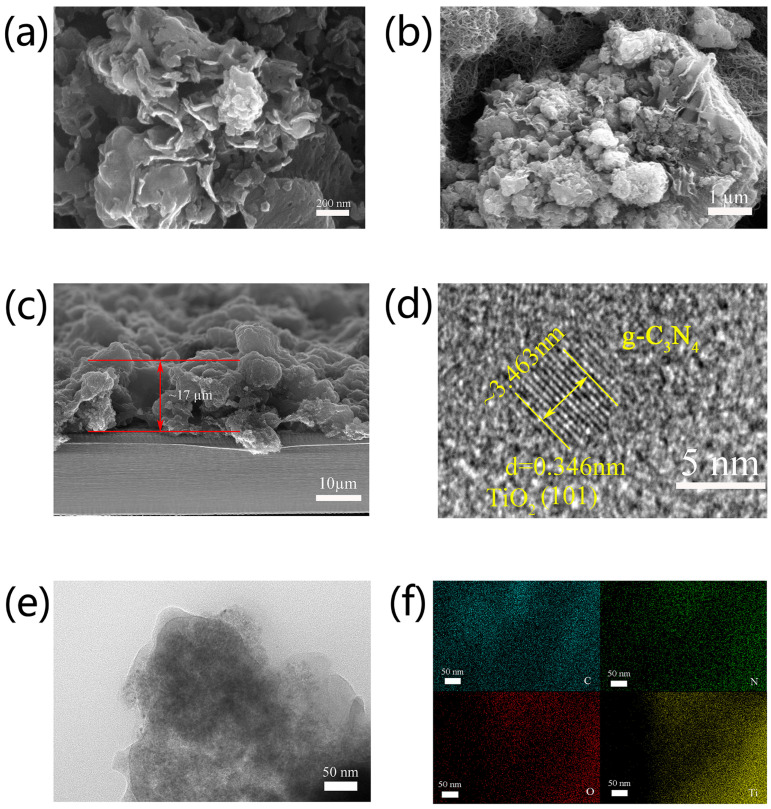
SEM images of (**a**) TiO_2_@g-C_3_N_4_, (**b**) TiO_2_@g-C_3_N_4_/CNT/PP, and (**c**) cross-sectional view of TiO_2_@g-C_3_N_4_/CNT/PP-modified separator. (**d**) HRTEM image, (**e**) TEM image, and (**f**) elemental mapping (C, N, O, and Ti) of TiO_2_@g-C_3_N_4_.

**Figure 2:**
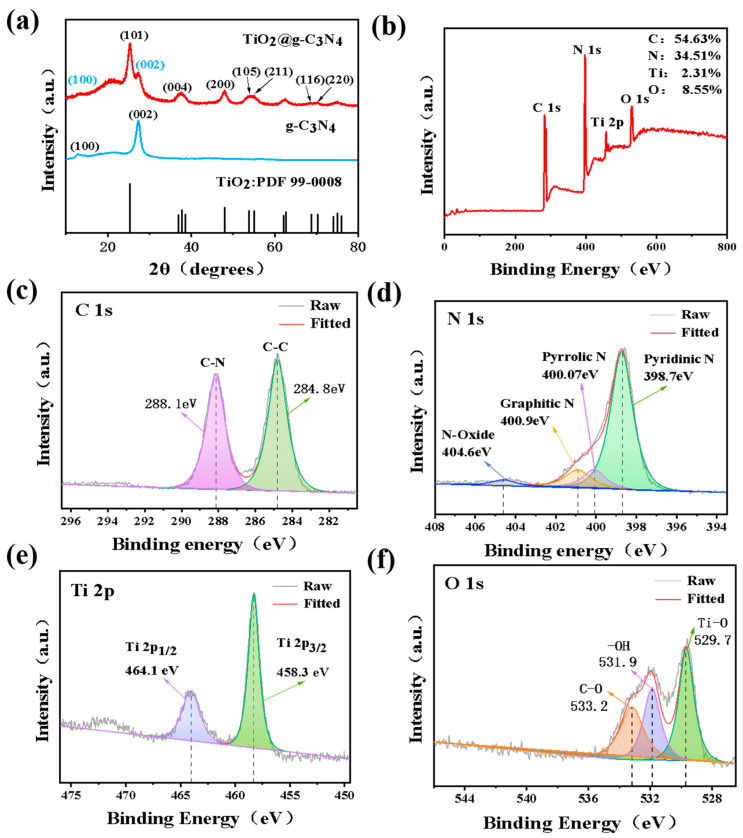
(**a**) XRD pattern of TiO_2_@g-C_3_N_4_. XPS spectra of TiO_2_@g-C_3_N_4_: (**b**) survey, (**c**) C 1s, (**d**) N 1s, (**e**) Ti 2p, and (**f**) O 1s spectra.

The microstructure of the TiO_2_@g-C_3_N_4_ composite was characterized by SEM and TEM. SEM images indicated that the prepared TiO_2_@g-C_3_N_4_ had a pleated thin layer structure (Figure 1a), and the surface of this pleated layer had abundant active sites. These sites could act as favorable adsorption catalytic sites for LiPSs. Furthermore, the pleated thin layer structure can provide additional mechanical cushioning space, so that it can recover after being bent. Figure 1b shows that TiO_2_@g-C_3_N_4_ was crisscrossed by the MWCNT conductive network, which partially compensated for the lack of conductivity within the TiO_2_@g-C_3_N_4_ composite. The addition of MWCNTs improved the electron/ion transfer at the electrode side, enabling the effective capture of LiPSs. Figure 1c shows that the thickness of the modified separator was 17 µm, and this thickness was uniformly distributed. A high-resolution TEM image of the composite is shown in Figure 1d. Bragg’s law (2dsinθ = nλ) was used to calculate the lattice fringe spacing (d = 0.346 nm, corresponding to the (101) plane of TiO_2_). This demonstrated that nano-TiO_2_ had successfully been loaded on the surface of the thin g-C_3_N_4_ layer. High-resolution TEM images further demonstrated the thin layer morphology of the composite, and the EDS analysis showed that TiO_2_ nanoparticles were uniformly distributed in the thin g-C_3_N_4_ layer (Figure 2e,f). This combination of 0D/2D morphology was beneficial for enabling good interfacial contact between the two materials.

The crystal structure of the composite was analyzed by XRD (Figure 2a). The diffraction peaks of TiO_2_@g-C_3_N_4_ at 25.5°, 37.8°, 48.0°, 55.1°, and 70.3° corresponded to the (101), (004), (200), (105), (211), and (220) planes of standard TiO_2_ (PDF 99-0008), respectively. The characteristic peaks at 13.3° and 27.6° corresponded to the (002) and (100) planes of g-C_3_N_4_. These sharp peaks indicated the good crystallinity of the composite and further demonstrated its successful preparation.

XPS was used to further understand the surface element states of the TiO_2_@g-C_3_N_4_ composite. As shown in Figure 2b, the full survey spectrum of TiO_2_@g-C_3_N_4_ indicated the presence of four elements: N, C, Ti, and O. The 54.63 at.% C content reflected the high specific gravity of g-C_3_N_4_. The C 1s spectrum (Figure 2c) showed two peaks at 284.8 eV and 288.1 eV that corresponded to C–C and C–N bonds, respectively. The N 1s spectrum (Figure 2d) showed four nitrogen sub-peaks. The peaks at 398.7 eV, 400.07 eV, 400.9 eV, and 404.6 eV corresponded to pyridine nitrogen, pyrrole nitrogen, graphite nitrogen, and nitrogen oxides, respectively. The structure of C–N heteroatoms can influence the electron distribution; therefore, the interface of the TiO_2_@g-C_3_N_4_ (N-n type) heterojunction with the C–N heteroatom has a strongly polar surface. Pyridine nitrogen has excellent affinity with LiPSs, enabling their adsorption. Thus, the high pyridine nitrogen content showed good promise for enhancing the sulfur fixation ability of TiO_2_@g-C_3_N_4_. The Ti 2p spectrum (Figure 2e) showed peaks at 458.3 eV and 464.1 eV corresponding to Ti 2p_1/2_ and Ti 2p_3/2_, respectively. The existence of some impurity peaks may be due to the low Ti atom content of only 2.31% in TiO_2_. The O 1s spectrum (Figure 2f) showed three peaks at 529.7 eV, 532.9 eV, and 533.2 eV, which corresponded to Ti–O bonds, –OH groups, and C–O bonds, respectively. These XPS results further demonstrated the effective preparation of TiO_2_@g-C_3_N_4_, and indicated the strong polarity of the composite surface. This provides a basis for the strong adsorption of polysulfide.

### 3.2. Mechanical Properties and Wettability

**Figure 3:**
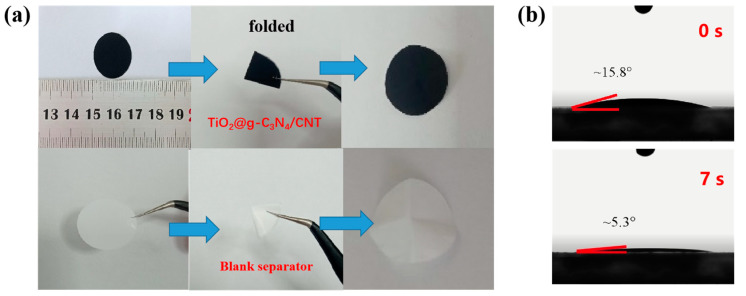
(**a**) Digital photograph of the TiO_2_@g-C_3_N_4_/CNT/PP-modified separator and blank PP separator during mechanical performance test; (**b**) electrolyte contact angle of the TiO_2_@g-C_3_N_4_/CNT/PP-modified separator at different times.

The mechanical properties and wettability of the separator had a direct impact on the overall performance of the battery. Figure 3a shows a comparison of the TiO_2_@g-C_3_N_4_-modified separator with a blank PP separator. Optical images show that the modified separator was able to recover after two large folds, with no observed material peeling or falling off. In contrast, the blank PP separator was noticeably deformed and could not be recovered. Therefore, the separator coated with the TiO_2_@g-C_3_N_4_ composite exhibited good adhesion and mechanical properties. In addition, the contact angle of the modified separator was measured to explore the electrolyte (1.0 M LiTFSI and 0.1 M LiNO_3_) wettability. The contact angle rapidly decreased by approximately 10° from 0 s to 7 s, as shown in Figure 3b. The rapid absorption and wettability of the electrolyte on the separator demonstrated the strong affinity of the TiO_2_@g-C_3_N_4_/CNT coating to the electrolyte.

### 3.3. Adsorption Test

**Figure 4:**
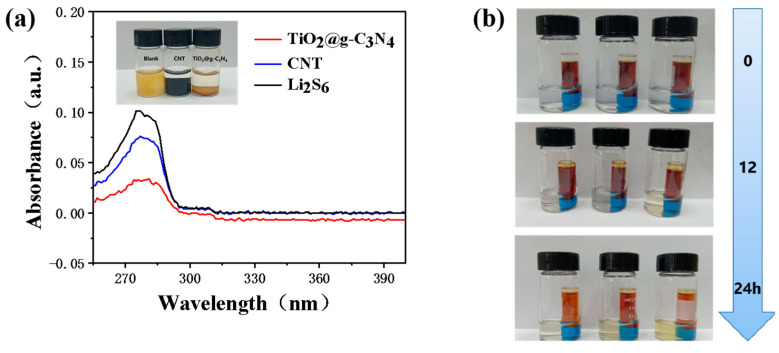
(**a**) UV–vis spectra of Li_2_S_6_ solutions containing TiO_2_@g-C_3_N_4_ or CNT (inset: photograph of solutions before and after adsorption for 3 h); (**b**) digital photographs showing the diffusion of Li_2_S_6_ across TiO_2_@g-C_3_N_4_/CNT/PP, TiO_2_/CNT/PP, and CNT/PP separators for 24 h.

In this study, the LiPSs adsorption properties of TiO_2_@g-C_3_N_4_ were verified by performing visible adsorption experiments with different materials. The same mass of CNT and TiO_2_@g-C_3_N_4_ was each added to separate Li_2_S_6_ solutions, which were thoroughly mixed and left for 3 h (Figure 4a inset). Compared with the orange color of the original Li_2_S_6_ solution, the Li_2_S_6_ supernatant obtained after adding TiO_2_@g-C_3_N_4_ was clear and transparent. The concentration of the Li_2_S_6_ supernatant was quantitatively analyzed by UV–vis spectroscopy (Figure 4a), which demonstrated the superior LiPSs adsorption capacity of TiO_2_@g-C_3_N_4_ compared with CNT. In addition, a permeation experiment was performed to evaluate the diffusion behavior of Li_2_S_6_ by observing the colors of Li_2_S_6_ solutions exposed to three different modified separators at 0 h, 12 h, and 24 h, as shown in Figure 4b. Clearly, only a small amount of Li_2_S_6_ passed through the TiO_2_@g-C_3_N_4_/CNT-modified separator after 24 h, which indicated that the modified separator had an excellent inhibitory effect on polysulfide diffusion. Figure S1 shows XPS spectra of TiO_2_@g-C_3_N_4_ obtained after the adsorption of Li_2_S_6_. Some new peaks (polysulfide, thiosulfate, etc.) were observed in the S 2p spectrum (Figure S1a), confirming the interaction between TiO_2_@g-C_3_N_4_ and Li_2_S_6_. In addition, the Li 1s spectrum (Figure S1b) showed the presence of Li-S and Li-N bonds, indicating the adsorption of Li_2_S_6_ by TiO_2_@g-C_3_N_4_. Therefore, in addition to demonstrating the effective preparation of TiO_2_@g-C_3_N_4_, XPS analysis indicated the strong adsorption of polysulfide on the composite surface.

### 3.4. Catalytic Performance and Electrochemical Test

**Figure 5:**
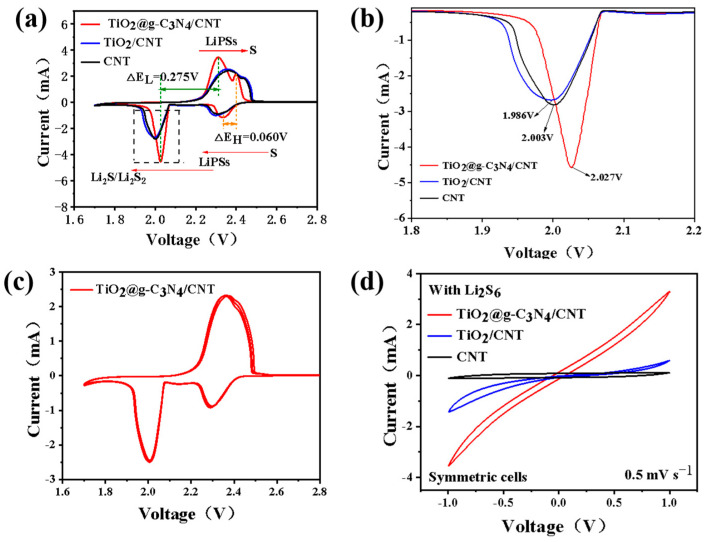
(**a**) CV curves of Li–S batteries prepared with TiO_2_@g-C_3_N_4_/CNT, TiO_2_/CNT, and CNT-modified separators; (**b**) magnified view of the large reduction peak from (**a**); (**c**) CV curves of TiO_2_@g-C_3_N_4_/CNT-modified separator obtained in three cycles at a scan rate of 0.1 mV s^−1^; (**d**) CV curves of symmetrical cells prepared with different electrodes.

To verify the actual performance of the modified separator in a lithium–sulfur battery, button cell batteries were assembled using CNT, TiO_2_/CNT, or TiO_2_@g-C_3_N_4_/CNT-modified separators. The CV curves of these cells were obtained at a scanning rate of 0.1 mV s^−1^, as shown in Figure 5a. In these curves, the small reduction peak corresponded to the reduction of S_8_ into Li_2_S_n_, the large reduction peak corresponded to the reduction of Li_2_S_n_ to insoluble Li_2_S/Li_2_S_2_, and the two oxidation peaks corresponded to the reverse of the reduction reaction. The battery prepared with the TiO_2_@g-C_3_N_4_/CNT-modified separator showed a higher reduction potential and lower oxidation potential, and its polarization potential of 60 mV was much smaller than that of the cells prepared with CNT and TiO_2_/CNT separators. A magnified view of the reduction peak (Figure 5b) shows that the battery prepared with the TiO_2_@g-C_3_N_4_/CNT-modified separator had the highest reduction potential of 2.027 V. Therefore, the construction of the TiO_2_@g-C_3_N_4_ heterojunction reduced the polarization voltage and significantly promoted the catalytic conversion of polysulfide. Three cyclic CV curves of these batteries (Figure 5c and Figure S2) showed that the battery assembled with the TiO_2_@g-C_3_N_4_/CNT-modified separator had highly overlapping curves and a higher redox peak current. Thus, this separator led to excellent reversibility of the conversion reaction and better kinetics compared with the other separators. To further verify the accelerated catalytic conversion of polysulfide by TiO_2_@g-C_3_N_4_, the TiO_2_@g-C_3_N_4_/CNT composite was assembled into a symmetrical battery. In the range of −1 to 1 V, CV curves (Figure 5d) showed that the TiO_2_@g-C_3_N_4_/CNT symmetrical battery had a higher current response. This demonstrated that the TiO_2_@g-C_3_N_4_ composite had very high electrocatalytic activity and a faster Li_2_S_n_ catalytic conversion rate.

**Figure 6:**
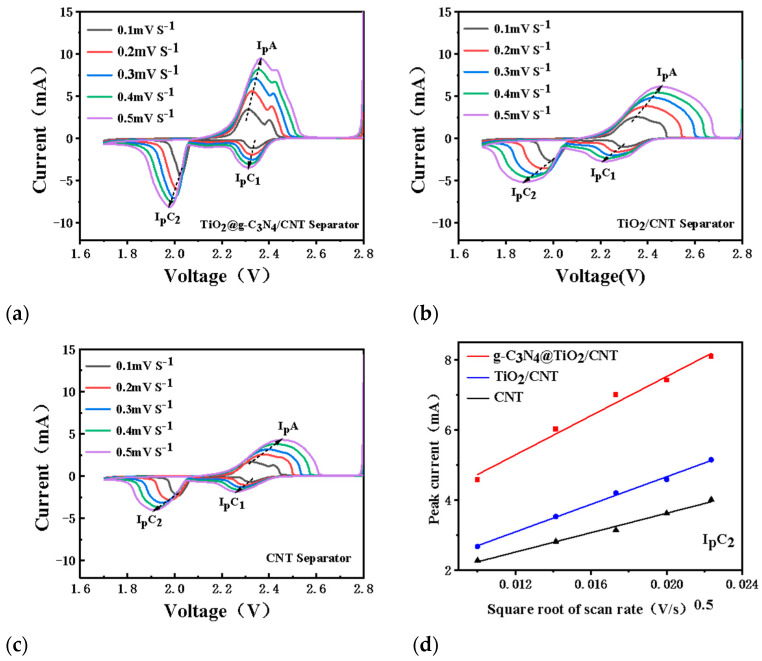

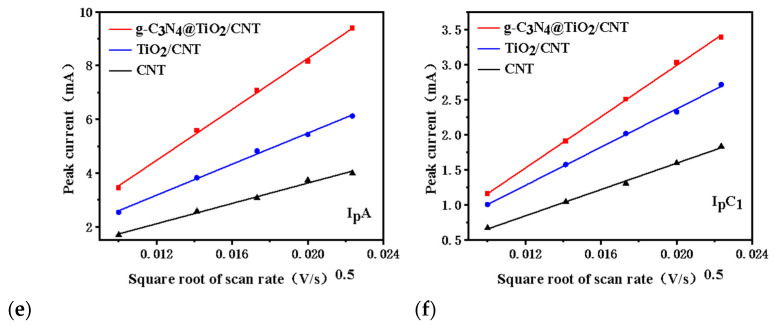
(**a**–**c**) CV curves of TiO_2_@g-C_3_N_4_/CNT, TiO_2/_CNT, and CNT cells in the range of 0.1–0.5 mV s^−1^; (**d**–**f**) dependence of the CV peak currents on the scan rate for the Li–S batteries assembled with different separators.

Figure 6a–c shows the CV curves of batteries prepared with the TiO_2_@g-C_3_N_4_/CNT, TiO_2_/CNT, and CNT-modified separators at different scanning speeds. These CV curves were obtained at 0.1–0.5 mV s^−1^, and the Li diffusion coefficient, *D_Li+_,* of the cells assembled with different modified separators were calculated as follows (Table S1) [24]:

$$I_{p}=\left(2.65\times 10^{5}\right)n^{1.5}AD_{Li^{+}}^{0.5}C_{Li^{+}}v^{0.5}$$

where *I_p_* is the peak current, n is the number of electrons involved in the reaction (for Li–S batteries, *n* = 2), *A* is the active electrode area (1.131 cm^−2^ in this study), *D_Li+_* is the *Li^+^* diffusion coefficient, *v* is the corresponding scanning rate, and *C_Li+_* is the *Li^+^* concentration in the electrolyte (1 M in this study).

Figure 6d–f shows the linear fitting between the peak current of the oxidation peak (*I_p_A*) and two reduction peaks (*I_p_*C_2_ and *I_p_*C_1_) with the square root of the scan rate. With the increasing scan rate, the peak current increased and the peak potential significantly shifted. The CV curve of the battery prepared with the TiO_2_@g-C_3_N_4_/CNT-modified separator showed a small peak potential shift, a large peak current change, and a large linear fitting slope. The slope of this CV curve was positively correlated with Li^+^ diffusion, indicating a typical diffusion-controlled process. Furthermore, the *D_Li+_* values of the different separators (Table S1) were calculated using the linear fitting slope values. Compared with the batteries prepared with the other separators, the battery with the TiO_2_@g-C_3_N_4_/CNT-modified separator had a higher *Li^+^* diffusion coefficient, and its *D_Li+_* values calculated using *I_p_*A, *I_p_*C_2_, and *I_p_*C_1_ were 3.03 × 10^−7^, 4.504 × 10^−8^, and 1.051 × 10^−7^, respectively. From a theoretical point of view, this further demonstrated that the battery assembled with the TiO_2_@g-C_3_N_4_/CNT-modified separator had better Li^+^ diffusion and migration performance. This was due to the strong adsorption and catalysis of LiPSs on the porous folded TiO_2_@g-C_3_N_4_.

**Figure 7:**
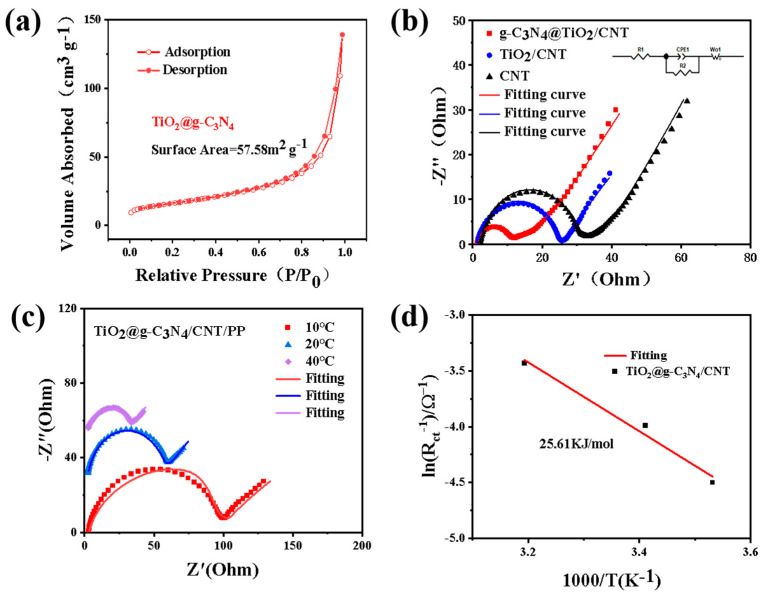
(**a**) Nitrogen adsorption–desorption isotherm of TiO_2_@g-C_3_N_4_; (**b**) electrochemical impedance spectra of lithium–sulfur batteries assembled with different separators. (**c**) Temperature-dependent Nyquist plots of TiO_2_@g-C_3_N_4_/CNT and (**d**) the Arrhenius linear fitting plot.

The specific surface area of the TiO_2_@g-C_3_N_4_ composite was investigated using its N_2_ adsorption–desorption isotherm curve (Figure 7a). The BET surface area of 57.58 m^2^ g^−1^ was much smaller than that of pure g-C_3_N_4_, which was potentially caused by the increase in the overall mass density of the composite due to the loading of TiO_2_ nanoparticles on the surface of g-C_3_N_4_. The composite exhibited a type IV isotherm, indicating its mesoporous structure. To further understand the catalytic effect of the modified separator on LiPSs and the Li^+^ conductivity of the TiO_2_@g-C_3_N_4_/CNT-modified separator, the AC impedance (EIS) of different separator batteries was evaluated (Figure 7b) and the Li^+^ conductivity of each corresponding separator was calculated (Table S2). The TiO_2_@g-C_3_N_4_/CNT/PP separator showed a charge transfer impedance of 10.47 Ω and Li^+^ conductivity of 0.2312 mS cm^−1^. Moreover, increasing the temperature resulted in lower impedance and higher Li^+^ conductivity (Figure 7c,d). These results demonstrate the rapid chemical kinetics, good conductivity, and excellent catalytic conversion performance of the battery assembled with the TiO_2_@g-C_3_N_4_/CNT-modified separator.

**Figure 8:**
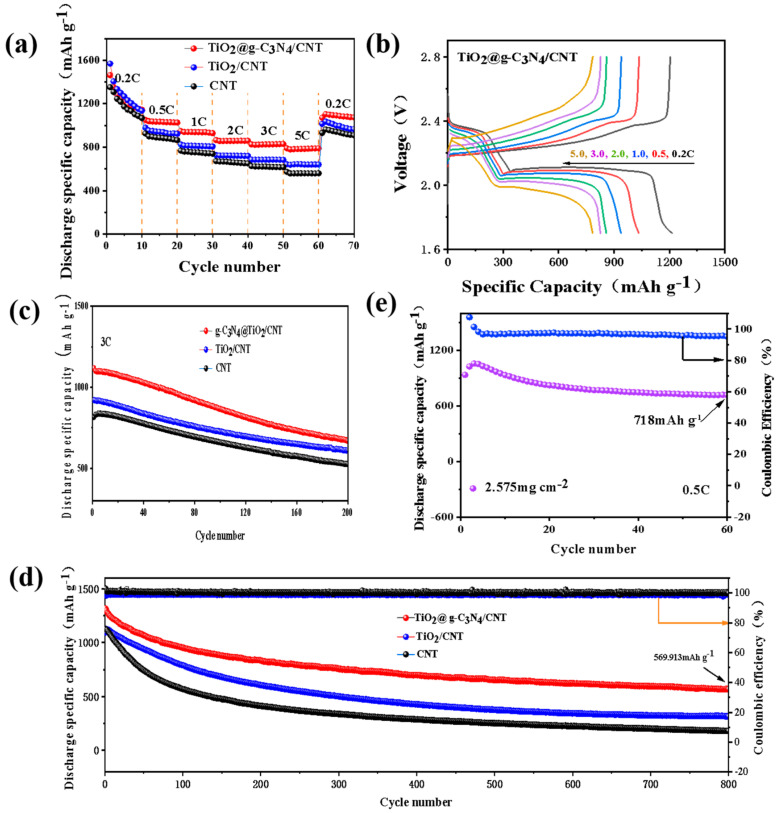
(**a**) Rate performance evaluation of Li–S batteries with different modified separators; (**b**) GCD curves of TiO_2_@g-C_3_N_4_/CNT/PP cells at different current densities; (**c**,**d**) long-term cycling at rates of 3 C and 1 C; (**e**) cycle performance of TiO_2_@g-C_3_N_4_/CNT/PP cell with a sulfur content of 2.58 mg cm^−2^ at 0.5 C.

The rate performance of batteries assembled with TiO_2_@g-C_3_N_4_/CNT, TiO_2_/CNT, and CNT-modified separators was evaluated at current densities of 0.2–5 C and the corresponding constant current charge–discharge curves (GCD) were obtained at different rates, as shown in Figure 8a,b and Figure S3. The battery assembled with the TiO_2_@g-C_3_N_4_/CNT-modified separator showed the highest rate performance and a clear charge–discharge platform. This battery had specific discharge capacities of 1463.1, 1051.9, 949.1, 865.3, 825.9, and 790.5 mAh g^−1^ at current densities of 0.2, 0.5, 1, 2, 3, and 5 C, respectively. When the current density returned to 0.2 C, a high discharge specific capacity of 1071.5 mAh g^−1^ was obtained. This demonstrated the excellent rate performance and redox reversibility of the modified separator. Moreover, the GCD curve showed the presence of a smooth charging and discharging platform even at a high current density of 5 C, and smaller polarization voltages were obtained at different rates. The excellent catalytic activity and rapid redox kinetics of the composite to LiPSs were further confirmed.

To further evaluate the cycle stability of the modified separator battery, long-term cycling was performed at 1 C and 3 C, and a high sulfur load test was also carried out. As shown in Figure 8c,d, compared with the other modified separators, the initial discharge specific capacity of the battery prepared with the TiO_2_@g-C_3_N_4_/CNT-modified separator at 1 C was 1316.4 mAh g^−1^, and after 800 cycles, a specific capacity of 569.9 mAh g^−1^ was retained. The cycle attenuation rate per cycle was only 0.07%, and a Coulombic efficiency of ~99% was achieved. Therefore, this battery had good cycle stability and a high utilization rate of active substances. Compared with many other reported results (Table S3), this battery exhibited good electrochemical cycle stability. In addition, at a higher rate of 3 C, a specific capacity of 669.9 mAh g^−1^ was retained after 200 cycles. These results further demonstrate that using TiO_2_@g-C_3_N_4_/CNT to modify the separator of a lithium–sulfur battery can significantly improve the conversion rate of LiPSs, activate dead sulfur, and inhibit the shuttle effect. As shown in Figure 8e, the potential of using the TiO_2_@g-C_3_N_4_/CNT-modified separator in a practical application was studied using a sulfur loading of 2.58 mg cm^−2^. After 60 cycles at 0.5 C, a specific capacity of 718 mAh g^−1^ was still maintained. This excellent performance can be attributed to the effective physical adsorption and catalysis of LiPSs on the rich active sites of the TiO_2_@g-C_3_N_4_ heterojunction composite. This result indicates the possibility of using this modified separator in practical lithium–sulfur battery applications.

## 4. Conclusions

In this study, a 0D/2D composite catalytic material with a heterogeneous structure was constructed through a simple heat treatment process. TiO_2_ nanoparticles were supported on the surface of a layered g-C_3_N_4_ structure, forming a large number of neutral active sites. This enabled the strong adsorption of LiPS intermediates and accelerated their catalytic conversion. Thus, the Li transmission rate in the lithium–sulfur battery was improved. In addition, CNT was introduced to construct a long-distance network-like electron transmission path to improve the poor electron conductivity of the separator. The experimental results showed that the TiO_2_@g-C_3_N_4_ composite has strong adsorption properties, good kinetics, high catalytic activity, and high Li^+^ conductivity for polysulfides. A lithium–sulfur battery prepared with a TiO_2_@g-C_3_N_4_/CNT-modified separator was able to retain a specific capacity of 569.9 mAh g^−1^ after 800 cycles at 1 C, and the cycle attenuation rate per cycle was only 0.07%. When the sulfur load was 2.58 mg cm^−2^, a high specific capacity of 718 mAh g^−1^ was retained after 60 cycles at 0.5 C. These results demonstrate that the construction of multifunctional heterostructures can effectively improve lithium-ion battery performance. However, there is still room for improvement of the cathode-side conductivity of the modified separator. The TiO_2_@g-C_3_N_4_/CNT composite prepared in this study is a simple, effective, and promising material for modifying the separator in lithium–sulfur batteries and enhancing their electrochemical performance. Thus, this material shows good promise for commercial application.

## Data Availability

The data presented in this article are available on request from the corresponding author.

## Supplementary Materials

The following supporting information can be downloaded at: https://www.mdpi.com/article/10.3390/nano13243084/s1, Figure S1: The XPS spectra of the composite after adsorption (a) S 2p; (b) Li 1s; Figure S2: CV curves of the TiO_2_/CNT/PP and CNT/PP cells at a scan rate of 0.1 mV s^−1^; Figure S3: Voltage profiles cycled at various current densities from 0.2 to 5.0 C of the TiO_2_/CNT/PP and CNT/PP cells; Table S1: The list of the calculated diffusion coefficient of Li^+^; Table S2: The Li+ conductivity of separators; Table S3: Comparison the Li–S battery performance in this study with the currently published results. References [13,14,15,21,22,23,25,26,27,28,29] are mentioned in the Supplementary Materials.
