# Supplementary material for: Rational Design of TiO2@g-C3N4/CNT Composite Separator for High Performance Lithium-Sulfur Batteries to Promote the Redox Kinetics of Polysulfide
Source: Nanomaterials (Basel). 2023 Dec 5;13(24):3084. doi: 10.3390/nano13243084 (PMC10745898; doi:10.3390/nano13243084)
Supplement: Supplementary file 1 [file nanomaterials-13-03084-s001.zip › nanomaterials-2597292-supplementary.pdf]

Supporting information

Rational Design of TiO_2@g-C_3N_4/CNT Composite Separator for High Performance Lithium-Sulfur Batteries to Promote the Redox Kinetics of Polysulfide

Lingling Dong [1], Wen Jiang [1], Kefeng Pan [1,*] and Lipeng Zhang [2,*]

1 School of Chemistry and Chemical Engineering, Shandong University of Technology, Zibo 255049, China
2 School of Materials and New Energy, South China Normal University, Shanwei 516600, China
* Correspondence: xiaopandy@126.com (K.P.); 20219207@m.scnu.edu.cn (L.Z.)

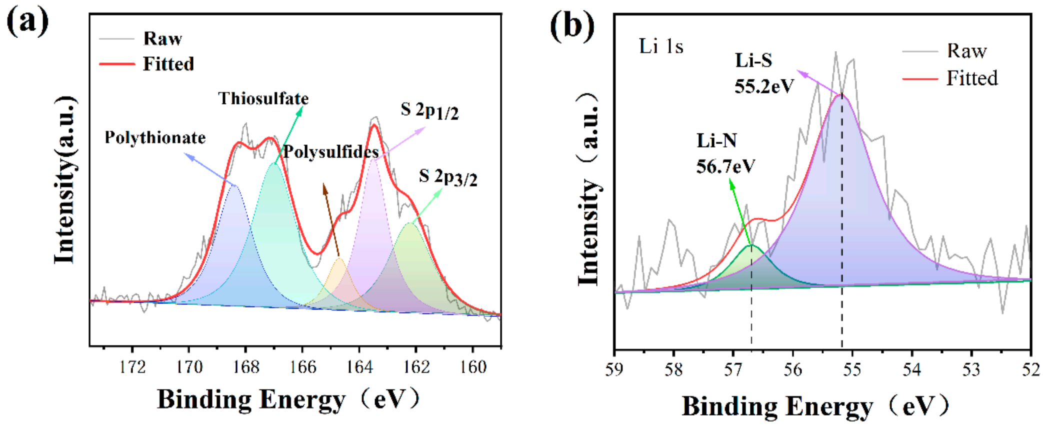

Figure S1.The XPS spectra of the composite after adsorption (a) S 2p; (b) Li 1s.

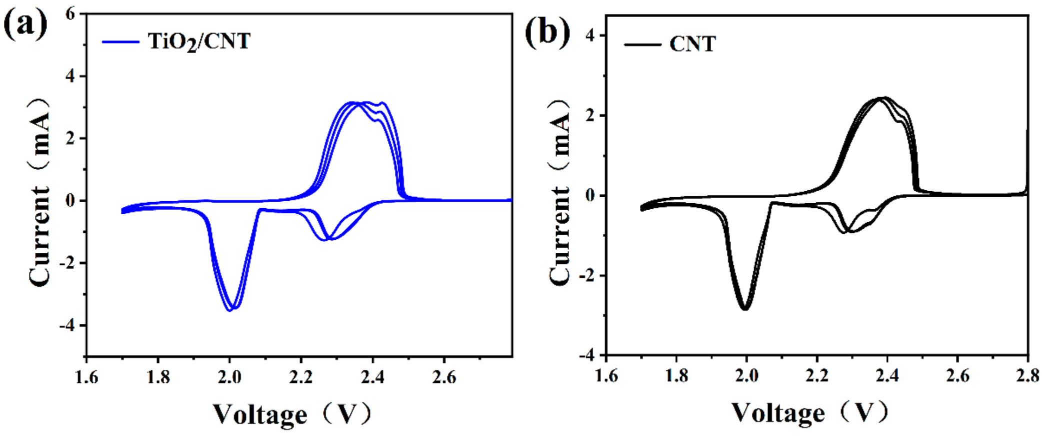

Figure S2.CV curves of the TiO_2/CNT/PP and CNT/PP cells at a scan rate of 0.1mV s^{-1}

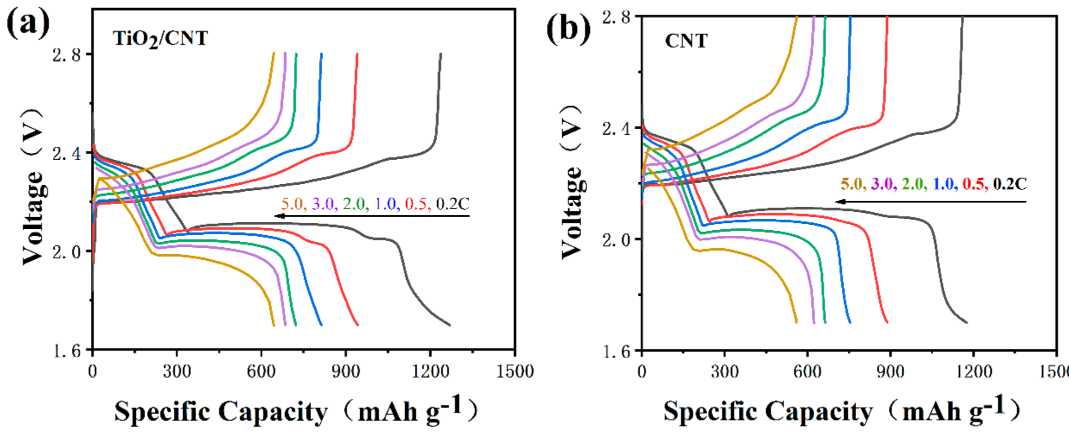

Figure S3. Voltage profiles cycled at various current densities from 0.2 to 5.0 C of the TiO_2/CNT/PP and CNT/PP cells

Table S1. The list of the calculated diffusion coefficient of Li^+.

D_{Li+} ($cm^2\ s^{-1}$)	I_A	I_{C1}	I_{C2}
g-C_3N_4@TiO_2/CNT	3.03×10^{-7}	4.504×10^{-8}	1.051×10^{-7}
TiO_2/CNT	1.13×10^{-7}	2.502×10^{-8}	5.198×10^{-8}
CNT	4.836×10^{-8}	1.185×10^{-8}	2.583×10^{-8}

Table S2.The Li^+ conductivity of separators

Separators	g-C_3N_4@TiO_2/CNT	TiO_2/CNT	CNT
Li^+ conductivity (~$mS\cdot cm^{-1}$)	0.2312	0.1156	0.0884

Table S3. Comparison the Li-S battery performance of this work with the currently published results.

Separator	initial discharge capacity	Rate	Electrochemical performance	Ref.
TiO_2@SCNT/PP	932 mAh g^{-1}	1 C	746.5mAh g^{-1} after 300 cycles	1
MWCNTs/CeO_2/PP	898.3 mAh g^{-1}	0.2 C	520.7 mAh g^{-1} after 300 cycles	2
TiO_2 NTs/RGO	1303.3 mAh g^{-1}	0.2 C	620.6 mAh g^{-1} after 100 cycles	3
Tg-C_3N_4/PP	1069 mAh g^{-1}	0.2 C	704 mAh g^{-1} after 100 cycles	4
g-C3N4/CNT@PP	~1000mAh g^{-1}	0.2 C	870.5 mAh g^{-1} after 100 cycles	5
NC/TiN NWs@PP	~1100mAh g^{-1}	1 C	720 mAh g^{-1} after 150 cycles	6
TiO_2 NT/CNT	936 mAh g^{-1}	1 C	557 mAh g^{-1} after 200 cycles	7
TiO_2/CNF	935 mAh g^{-1}	1 C	693.8 mAh g^{-1} after 500 cycles	8
PTCN/S	810 mAh g^{-1}	1 C	553 mAh g^{-1} after 500 cycles	9
TiO_2 NS/CNT	1247 mAh g^{-1}	0.2 C	627mAh g^{-1} after 100 cycles	10
HPCA-TO@PP	~850mAh g^{-1}	1 C	513mAh g^{-1} after 200 cycles	11
g-C_3N_4@TiO_2/CNT/PP	1316mAh g^{-1}	1 C	834.2 mAh g^{-1} after 200 cycles	This work

References

1. Gao, Z.; Xue, Z.; Miao, Y.; Chen, B.; Xu, J.; Shi, H.; Tang, T.; Zhao, X. TiO_2@Porous carbon nanotubes modified separator as polysulfide barrier for lithium-sulfur batteries. *J. Alloys Compd.* **2022**, *906*, 164249.
2. Zhu, W.; Zhang, Z.; Wei, J.; Jing, Y.; Guo, W.; Xie, Z.; Qu, D.; Liu, D.; Tang, H.; Li, J. A synergistic modification of polypropylene separator toward stable lithium–sulfur battery. *J. Membr. Sci.* **2020**, *597*, 117646.
3. Gui, Y.; Chen, P.; Liu, D.; Fan, Y.; Zhou, J.; Zhao, J.; Liu, H.; Guo, X.; Liu, W.; Cheng, Y. TiO_2 nanotube/RGO modified separator as an effective polysulfide-barrier for high electrochemical performance Li-S batteries. *J. Alloys Compd.* **2022**, *895*, 162495.
4. Liu, X.; Ma, H.; Hu, C.; Liu, N.; Zhao, Y. Tg-C_3N_4-coated functional separator as polysulfide barrier of high-performance lithium-sulfur batteries. *Nanotechnology* **2021**, *32*, 475401.
5. Wang, X.; Li, G.; Li, M.; Liu, R.; Li, H.; Li, T.; Sun, M.; Deng, Y.; Feng, M.; Chen, Z. Reinforced polysulfide barrier by g-C_3N_4/CNT composite towards superior lithium-sulfur batteries. *J. Energy Chem.* **2021**, *53*, 234–240.
6. Kim, Y.; Noh, Y.; Bae, J.; Ahn, H.; Kim, M.; Kim, W.B. N-doped carbon-embedded TiN nanowires as a multifunctional separator for Li–S batteries with enhanced rate capability and cycle stability. *J. Energy Chem.* **2021**, *57*, 10–18.
7. Chen, A.; Liu, W.; Yan, J.; Liu, K. A novel separator modified by titanium dioxide nanotubes/carbon nanotubes composite for high performance lithium-sulfur batteries. *Funct. Mater. Lett.* **2019**, *12*, 1950016.
8. Liang, G.; Wu, J.; Qin, X.; Liu, M.; Li, Q.; He, Y.-B.; Kim, J.-K.; Li, B.; Kang, F. Ultrafine TiO_2 Decorated Carbon Nanofibers as Multifunctional Interlayer for High-Performance Lithium-Sulfur Battery. *ACS Appl. Mater. Interfaces* **2016**, *8*, 23105–23113.
9. Ma, H.; Liu, X.; Liu, N.; Zhao, Y.; Zhang, Y.; Bakenov, Z.; Wang, X. Defect-rich porous tubular graphitic carbon nitride with strong adsorption towards lithium polysulfides for high-performance lithium-sulfur batteries. *J. Mater. Sci. Technol.* **2022**, *115*, 140–147.
10. Chen, P.; Wang, Z.; Zhang, B.; Zhao, J.; Liu, H.; Guo, X.; Liu, W.; Su, Z. Multi-functional TiO_2 nanosheets/carbon nanotubes modified separator enhanced cycling performance for lithium-sulfur batteries. *Int. J. Energy Res.* **2020**, *44*, 3231–3240.
11. Shi, C.; Huang, J.; Tang, Y.; Cen, Z.; Wang, Z.; Liu, S.; Fu, R. A hierarchical porous carbon aerogel embedded with small-sized TiO_2 nanoparticles for high-performance Li–S batteries. *Carbon* **2022**, *202*, 59–65.
